# Factors associated with duration of breastfeeding in women giving birth for the first time

**DOI:** 10.1186/s12884-022-05038-7

**Published:** 2022-09-22

**Authors:** David M. Haas, Ziyi Yang, Corette B. Parker, Judith Chung, Samuel Parry, William A. Grobman, Brian M. Mercer, Hyagriv N. Simhan, Robert M. Silver, Ronald J. Wapner, George R. Saade, Philip Greenland, Noel Bairey Merz, Uma M. Reddy, Victoria L. Pemberton, for the nuMoM2b study and the nuMoM2b Heart Health Study

**Affiliations:** 1 Department of OB/GYN, Indiana University School of Medicine, 550 N. University Blvd, UH 2440, Indianapolis, IN 46202 USA; 2 RTI International, Research Triangle, USA; 3 University of California, Irvine, USA; 4 University of Pennsylvania, Philadelphia, USA; 5 Northwestern University, Chicago, Illinois USA; 6 Case Western Reserve University, Cleveland, USA; 7 University of Pittsburgh, Pittsburgh, USA; 8 University of Utah, Salt Lake City, USA; 9 Columbia University, New York, USA; 10 University of Texas Medical Branch, Galveston, TX USA; 11 Smidt Cedars-Sinai Heart Institute, Los Angeles, CA USA; 12 Eunice Kennedy Shriver National Institute of Child Health and Human Development (NICHD), Bethesda, USA; 13 National Heart, Lung, and Blood Institute, NIH, Bethesda, USA

**Keywords:** Breastfeeding, Longevity, Nulliparous patients

## Abstract

**Objective:**

To examine maternal, psychosocial, and pregnancy factors associated with breastfeeding for at least 6 months in those giving birth for the first time.

**Methods:**

We performed a planned secondary analysis of an observational cohort study of 5249 women giving birth for the first time. Women were contacted at least 6 months after delivery and provided information regarding breastfeeding initiation, duration, and exclusivity. Maternal demographics, psychosocial measures, and delivery methods were compared by breastfeeding groups.

**Results:**

4712 (89.8%) of the women breastfed at some point, with 2739 (58.2%) breastfeeding for at least 6 months. Of those who breastfed, 1161 (24.7% of the entire cohort), breastfed exclusively for at least 6 months. In the multivariable model among those who ever breastfed, not smoking in the month prior to delivery (adjusted odds ratio [aOR] 2.04, 95%CI 1.19–3.45), having a Master’s degree of higher (aOR 1.89, 95%CI 1.51–2.36), having a planned pregnancy (aOR 1.48, 95%CI 1.27–1.73), older age (aOR 1.02, 95% CI, 1.01–1.04), lower BMI (aOR 0.96 95% CI 0.95–0.97), and having less anxiety measured during pregnancy (aOR 0.990, 95%CI 0.983–0.998) were associated with breastfeeding for at least 6 months. Compared to non-Hispanic White women, Hispanic women, while being more likely to breastfeed initially (aOR 1.40, 95%CI 1.02–1.92), were less likely to breastfeed for 6 months (aOR 0.72, 95%CI 0.59–0.88). While non-Hispanic Black women were less likely than non-Hispanic White women to initiate breastfeeding (aOR 0.68, 95%CI 0.51–0.90), the odds of non-Hispanic Black women of continuing to breastfeed for at least 6 months was similar to non-Hispanic White women (aOR 0.92, 95%CI 0.71–1.19).

**Conclusions:**

In this cohort of women giving birth for the first time, duration of breastfeeding was associated with several characteristics which highlight groups at greater risk of not breastfeeding as long as currently recommended.

**Trial registration:**

NCT01322529 (nuMoM2b) and NCT02231398 (nuMoM2b-Heart Health)

**Supplementary Information:**

The online version contains supplementary material available at 10.1186/s12884-022-05038-7.

## Introduction

The longer a woman breastfeeds her infant, the greater the benefits for both her own and her child’s health. Breastfed infants have decreased risk of infections, including gastrointestinal diseases, sepsis, wheezing respiratory tract infections, necrotizing enterocolitis, meningitis, and urinary tract infections [1–4]. Duration of being breastfed also is positively associated with intelligence later in life [5], as well as decreased prevalence of hypertension, hyperlipidemia, cardiovascular disease, diabetes and non-alcoholic fatty liver disease [6–9]. Indeed, excess deaths of women due to myocardial infarction, breast cancer, and diabetes have been attributed to lack of or a shorter duration of breastfeeding [10].

So powerful are the overall health benefits of breastfeeding that the World Health Organization’s fifth Global Nutrition Target for 2025 is to increase the rate of exclusive breastfeeding in the first 6 months to at least 50% of all mothers [11]. As of 2017, however, only 25.6% of US women were exclusively breastfeeding at 6 months, with only 58.3% doing any breastfeeding at 6 months [12]. Previous successful breastfeeding is a factor strongly associated with duration of breastfeeding for a subsequent child [13, 14]. However, for women giving birth for the first time who have not had the opportunity to breastfeed previously, it is important to determine other factors associated with breastfeeding duration to inform potentially effective interventions that could be used to improve that rate. Prior reviews found that not smoking, having a vaginal delivery, high maternal educational attainment, and specific breastfeeding education were associated with higher rates of breastfeeding continuation [13]. These factors might inform multilevel strategies to optimize conditions so that all individuals who wanted to breastfeed would be able to breastfeed for as long as they desired. Additionally, there is a gap in the literature as most previous work on breastfeeding duration did not include validated psychosocial measures as covariates. As much of the previous work is not generalizable to US populations, and given demographic trends in the United States, contemporary understanding of these factors in a large representative diverse cohort is needed.

Therefore, the objective of this study was to examine maternal (both demographic and psychosocial) and pregnancy factors associated with breastfeeding for at least 6 months in a cohort of women giving birth for the first time.

## Methods

### Participants and measures

This study was a planned secondary analysis of a large prospective cohort study in women pregnant with their first child. The “Nulliparous Pregnancy Outcomes Study: monitoring mothers-to-be” (nuMoM2b) project recruited 10,038 nulliparous women with singleton pregnancies from eight U.S. medical centers between 2010 and 2013 with the objective of identifying risk factors for and predictors of adverse pregnancy outcomes. Detailed methods of the nuMoM2b study are reported elsewhere [15]. The nuMoM2b Heart Health Study (HHS) followed 7003 women in the nuMoM2b cohort with interval contacts and an in-person study visit for cardiovascular health outcomes [16, 17]. Interval contacts for HHS began in 2013 and have been ongoing since then. Both the nuMoM2b and HHS studies were approved by the local Institutional Review Boards at each site and all women provided informed consent. Both studies were registered on clinicaltrials.gov (NCT01322529, NCT02231398).

In brief, women in the nuMoM2b cohort were recruited in the first trimester and had study visits in the 1st (Visit 1: gestational age 6 weeks 0 days to 13 weeks 6 days), 2nd (Visit 2: gestational age 16 weeks 0 days to 21 weeks 6 days), and late 2nd-early 3rd (Visit 3: gestational age 22 weeks 0 days to 29 weeks 6 days) trimesters, and at the time of delivery (Visit 4). During study visits, which were conducted in English or Spanish, multiple questionnaires and psychosocial instruments were completed and biological specimens were obtained [15]. Psychosocial factors evaluated included: depression (Edinburgh Perinatal Depression Scale (EPDS), Visit 3) [18], perceived stress (Cohen Perceived Stress Scale (PSS), Visit 1) [19], social support (Multidimensional Scale of Perceived Social Support, Visit 1) [20], perceived anxiety (Spielberger Trait Anxiety Subscale, Visit 1) [21], resilience (Connor-Davidson Resilience Scale, Visit 2) [22] and perceived pregnancy experience (Pregnancy Experience Scale (PES), Visit 3) [23]. Characteristics of the psychosocial measures in the overall cohort have been presented elsewhere [24, 25]. Race and ethnicity were self-reported and collected in standard categories as required by the Federal funding agency (https://obamawhitehouse.archives.gov/omb/fedreg_1997standards). After delivery, certified chart abstractors extracted delivery outcomes from the medical records. During the postpartum stay, the method of feeding the newborn at discharge (breastfeeding, formula feeding, or a combination of both) was also abstracted.

For the HHS, interviews were performed by telephone or email at 6-month intervals, beginning at least 6 months after delivery of the index pregnancy. Interviews were conducted in English or Spanish. During interviews, women updated contact information and answered questionnaires regarding any subsequent pregnancies, medications, medical conditions, cardiovascular events or diagnoses, and the health of their child. In addition, during the first interview, they answered questions about how their child was fed since delivery (Supplementary file 1). Specifically, women were asked if they ever breastfed their baby. If they responded “yes”, they were further asked how long their child was exclusively fed breast milk (“Was there a period of time when you fed this baby only breast milk (no formula, milk, juice, or food)? This is called exclusive breastfeeding.”), and how old the infant was when exclusive (“About how old was this baby when exclusive breastfeeding stopped?”) and non-exclusive breastfeeding (“About how old was this baby when all breastfeeding stopped?”) were discontinued [17]. Discontinuation options included: “Less than 6 weeks”; “6 weeks to 11 weeks”; “3–6 months”; “More than 6 months”; “Still breastfeeding”; and “Don’t know.” Women were considered to be breastfeeding for at least 6 months if they responded, “More than 6 months” or “Still breastfeeding.” The questions on breastfeeding were only asked at the first interval contact and were self-reported.

### Analysis

Descriptive statistics were used to describe participant characteristics and psychosocial scales according to whether women claimed ever breastfeeding, breastfeeding for at least 6 months, and exclusively breastfeeding for at least 6 months. Due to the response options being in the categories listed above, breastfeeding duration was not analyzed as a continuous variable. Pairwise comparisons were conducted using a Student’s t-test for normally distributed continuous variables, the Wilcoxon-rank sum test for non-normally distributed continuous variables, and the chi-square test for categorical variables.

Multivariable logistic regression was then performed for the outcome of ever breastfeeding and the two conditional outcomes of any breastfeeding for at least 6 months and exclusive breastfeeding for at least 6 months. We included variables in the model that were significantly different (*p* < 0.05) for each outcome in the univariate analysis. Characteristics present in < 1% of the cohort were excluded from the regression. All analyses were conducted in SAS v9.4 (SAS Institute Inc., Cary, NC).

## Results

### Overall cohort

Of the original 10,038 nuMoM2b participants, 8838 were eligible for contacting in the HHS. Of those women, 7003 (79.2%) women were successfully contacted for HHS, with 5249 (74.9%) of them having complete data for all questions and making up the analytic cohort for this study. Characteristics of the cohort are presented in Table 1. A total of 4712 (89.8%) women claimed to have breastfed their infant at some point. Women who ever breastfed tended to be older, with lower BMI, and were more often non-Hispanic White, higher earning category, and had higher educational attainment (all *p* < 0.001). Additionally, women who ever breastfed were more likely to state that the pregnancy was planned (67.7% vs 41.3%, *p* < 0.001) and were less likely to have smoked in the month prior to delivery (1.9% vs 10.4%, *p* < 0.001). Women who delivered vaginally were statistically more likely to breastfeed their infants than women delivered by cesarean, although the absolute difference was small (90.3% vs 88.2%, *p* = 0.02).

**Table 1:** Descriptive statistics for breastfeeding outcomes in the cohort

Variable	Overall ***N*** = 5249	Did you ever breastfeed this baby? ***N*** = 5249			How old was this baby when all breastfeeding stopped? ***N*** = 4704 (8 missing)			How old was this baby when exclusive breastfeeding stopped? ***N*** = 3726 (1 missing)		
		Yes ***N*** = 4712	No ***N*** = 537	P*	> 6 m ***N*** = 2739	<=6 m ***N*** = 1965	P*	> 6 m ***N*** = 1161	<=6 m ***N*** = 2565	P*
**Age, mean (SD)**	27.6 (5.4)	27.9 (5.)	24.9 (6.0)	< 0.001	28.8 (4.8)	26.6 (5.6)	< 0.001	28.8 (4.7)	27.6 (5.3)	< 0.001
**BMI, mean (SD)**	26.2 (6.2)	25.9 (5.9)	29.2 (8.3)	< 0.001	25.1 (5.1)	26.9 (6.6)	< 0.001	25.0 (4.9)	25.8 (6.0)	< 0.001
**Race or Ethnicity**				< 0.001			< 0.001			0.32
Non-Hispanic white	3551 (67.7%)	3270 (69.4%)	281 (52.3%)		2073 (75.7%)	1192 (60.7%)		856 (73.7%)	1835 (71.5%)	
Non-Hispanic black	524 (10.0%)	377 (8.0%)	147 (27.4%)		149 (5.4%)	226 (11.5%)		75 (6.5%)	203 (7.9%)	
Hispanic	733(14.0%)	657 (13.9%)	76 (14.2%)		275 (10.0%)	382 (19.4%)		130 (11.2%)	312 (12.2%)	
Other	441 (8.4%)	408 (8.7%)	33 (6.2%)		242 (8.8%)	165 (8.4%)		100 (8.6%)	215 (8.4%)	
**Poverty**				< 0.001			< 0.001			< 0.001
> 200% of FPL	3317 (63.2%)	3136 (66.6%)	181 (33.7%)		2065 (75.4%)	1068 (54.4%)		867 (74.7%)	1697 (66.2%)	
100–200% of FPL	613 (11.7%)	546 (11.6%)	67 (12.5%)		291 (10.6%)	255 (13.0%)		134 (11.5%)	281 (11.0%)	
< 100% of FPL	550 (10.5%)	439 (9.3%)	111 (20.8%)		197 (7.2%)	241 (12.3%)		75 (6.5%)	259 (10.1%)	
Refused	769 (14.7%)	591 (12.5%)	170 (33.2%)		186 (6.8%)	401 (20.4%)		85 (7.3%)	328 (12.8%)	
**Education**				< 0.001			< 0.001			< 0.001
High school or less	168 (32.1%)	1336 (28.4%)	351 (65.4%)		522 (19.1%)	808 (41.1%)		239 (20.6%)	743 (29.0%)	
Bachelor’s degree or less	2167 (41.3%)	2035 (43.2%)	132 (24.6%)		1256 (45.9%)	779 (39.6%)		521 (44.9%)	1102 (43.0%)	
Master’s degree and higher	1395 (26.6%)	1341 (28.5%)	54 (10.1%)		961 (35.1%)	378 (19.2%)		401 (34.5%)	720 (28.1%)	
**Was this pregnancy planned, yes**	3410 (65.0%)	3188 (67.7%)	222 (41.3%)	< 0.001	2106 (76.9%)	1078 (54.9%)	< 0.001	882 (76.0%)	1723 (67.2%)	< 0.001
**Was this delivery by Cesarean, yes**	1418 (27.0%)	1251 (26.6%)	167 (31.1%)	0.02	683 (24.9%)	566 (28.8%)	0.003	298 (25.7%)	620 (24.2%)	0.33
**Did you smoke any tobacco products in the month before delivery? yes**	143 (2.7%)	87 (1.9%)	56 (10.4%)	< 0.001	20 (0.7%)	66 (3.4%)	< 0.001	7 (0.6%)	70 (2.7%)	< 0.001
**Gestational age at the time of delivery, mean (SD)**	38.9 (2.0)	39.0 (2.0)	38.4 (2.5)	< 0.001	39.1 (1.8%)	38.8 (2.2%)	< 0.001	39.1 (1.5)	39.0 (2.2)	0.53
**Mental health disorder, yes**	1164 (22.2%)	1031 (21.9%)	133 (24.8%)	0.13	574 (21.0%)	454 (23.1%)	0.08	244 (21.0%)	572 (22.3%)	0.38
**Depression, yes**	750 (14.3%)	671 (14.2%)	79 (14.7%)	0.77	378 (13.8%)	292 (14.9%)	0.31	165 (14.2%)	353 (13.8%)	0.71
**Bipolar, yes**	79 (1.5%)	58 (1.2%)	21 (3.9%)	< 0.001	17 (0.6%)	40 (2.0%)	< 0.001	9 (0.8%)	40 (1.6%)	0.05
**Anxiety, yes**	593 (11.3%)	544 (11.5%)	49 (9.1%)	0.09	302 (11.0%)	241 (12.3%)	0.19	116 (10.0%)	311 (12.1%)	0.06

*m* months, *BMI* body mass index, *FPL* federal poverty level

*P-values obtained from t-test or chi-square test

Of the women who ever breastfed, 2739 (58.2%) continued breastfeeding for at least 6 months, with 1161 (24.7%) doing so exclusively. The same sociodemographic characteristics and directionality that were associated with “ever breastfeeding” above were also associated with “any breastfeeding for at least 6 months” among women who ever breastfed. Exclusive breastfeeding for at least 6 months was associated with older age, lower BMI, having higher income, having higher educational attainment, and not smoking (Table 2).

**Table 2:** Descriptive statistics for behavioral scales and associations with breastfeeding outcomes in the cohort

Variable	Overall ***N*** = 5249	Did you ever breastfeed this baby? ***N*** = 5249			How old was this baby when all breastfeeding stopped? ***N*** = 4704 (8 missing)			How old was this baby when exclusive breastfeeding stopped? ***N*** = 3726 (1 missing)		
		Yes ***N*** = 4712	No ***N*** = 537	***P***-Val*	> 6 m ***N*** = 2739	<=6 m ***N*** = 1965	***P***-Val*	> 6 m ***N*** = 1161	<=6 m ***N*** = 2565	***P***-Val*
**EPDS** Mean (SD) [Range 0–27. Higher values = more negative feelings/experiences]	5.5 (4.0)	5.4 (3.9)	6.6 (4.9)	< 0.001	5.0 (3.7)	5.9 (4.1)	< 0.001	5.0 (3.7)	5.3 (3.9)	0.01
**MSS** Mean (SD) [Range 12–84. Higher values = higher agreement]	74.7 (13.8)	75.1 (13.6)	71.4 (15.7)	< 0.001	76.0 (12.9)	73.9 (14.3)	< 0.001	76.3 (12.4)	74.9 (14.1)	0.002
**CD-RISC** Mean (SD) [Range 19–100. Higher values = Higher Resilience]	79.1 (11.4)	79.4 (11.0)	76.6 (13.5)	< 0.001	80.1 (10.6)	78.5 (11.5)	< 0.001	80.6 (10.8)	79.5 (10.9)	0.002
**STAI-T** Mean (SD) [Range 20–74. Higher values = Higher anxiety]	33.8 (8.6)	33.6 (8.4)	36.4 (9.5)	< 0.001	32.9 (8.0)	34.5 (8.8)	< 0.001	32.7 (8.0)	33.5 (8.4)	0.01
**PSS** Mean (SD) [Range 0–40. Higher values = Higher stress]	11.3 (6.3)	11.1 (6.2)	13.2 (7.1)	< 0.001	10.4 (5.7)	12.0 (6.6)	< 0.001	10.3 (5.9)	11.0 (6.2)	< 0.001
**PES** Median (Range)										
**PES-Hassle Frequency ratio**	0.70 (0.10–7.00)	0.70 (0.10–6.00)	0.70 (0.1–7.00)	0.01**	0.70 (0.10–6.00)	0.70 (0.1–3.00)	0.02**	0.70 (0.10–2.25)	0.70 (0.10–4.00)	0.76**
**PES-Hassle Intensity ratio**	0.56 (0.33–2.22)	0.56 (0.33–2.22)	0.60 (0.33–2.11)	< 0.001**	0.56 (0.33–2.04)	0.58 (0.33–2.22)	< 0.001**	0.56 (0.33–1.80)	0.56 (0.33–2.22)	0.31**

*EPDS* Edinburgh Postnatal Depression Scale, *MSS* Multidimensional Perceived Social Support Scale, *CD-RISC* Connor-Davidson Resilience Scale, *STAI-T* Spielberger State-Trait Anxiety Inventory, *PSS* Perceived Stress Scale, *PES* Pregnancy Experience Scale

*P-values obtained from t-test

**P-values obtained from Wilcoxon rank sum test

### Psychosocial associations

Psychosocial measures were also significantly different between women in different breastfeeding categories (Table 2). In general, women who had lower depression scores, higher social support scores, higher resiliency scores, lower anxiety measures, and lower perceived stress scores breastfed longer. The median scores for the pregnancy experiences hassle intensity ratio were lower for women who breastfed longer.

Psychosocial measures noted above which were associated with ever breastfeeding, breastfeeding for at least 6 months, or exclusive breastfeeding for at least 6 months individually (Table 2) were found not to be statistically significant when included in a logistic regression model together. As the psychosocial measures were found to have significant collinearity with one another (Pearson correlation coefficients available in Appendix), we included each in the logistic regression model one at a time and selected the measure most highly associated with the outcome to include in the final model. Given the strong association of the psychosocial measures with each other (Table 4 in Appendix), the Spielberger State-Trait Anxiety Inventory measure was selected to be included in the final regression model as it demonstrated the strongest association with the outcome.

### Logistic regression for associations with breastfeeding and duration

The final logistic regression (Table 3) demonstrated that women had higher odds of ever breastfeeding if they were Hispanic women (compared with non-Hispanic White women) (OR 1.40, 95% CI 1.02–1.92), had higher incomes (compared to < 100% of FPL- OR 1.59, 95% CI 1.12–2.27 for > 200% of FPL; OR 1.47, 95% CI 1.03–2.10 for 100–200% of FPL), higher educational attainment (compared to high school or less- OR 2.81, 95% CI 1.90–4.14 for Master’s degree or higher; OR 2.12, 95% CI 1.62–2.76 for bachelor degree or less), and had not smoked in the month prior to delivery (OR 2.32, 95% CI 1.56–3.45). Compared with non-Hispanic White women, non-Hispanic Black women had lower odds of breastfeeding their infant (OR 0.68, 95% CI 0.51–0.91), as did women with higher BMI (OR 0.95, 95% CI 0.94–0.97), and higher anxiety (OR 2.32, 95% CI 1.56–3.45 for each point increase on the scale).

**Table 3:** Logistic Regression associations of characteristics with breastfeeding outcomes

Variable	Did you ever breastfeed this baby Yes Vs No (reference group) ***N*** = 5249 Yes = 4712 No = 537			How old was this baby when all breastfeeding stopped? > 6 months Vs < = 6 months (reference) ***N*** = 4704 > 6 m = 2739 < =6 m = 1965			How old was this baby when exclusive breastfeeding stopped? > 6 months Vs <= 6 months (reference) ***N*** = 3726 > 6 m = 1161 < =6 m = 2565		
	Model AUC = 0.79 (0.76–0.81), ***N*** = 5249			Model AUC = 0.71 (0.69–0.72), ***N*** = 4704			Model AUC = 0.59 (0.57–0.61), ***N*** = 3726		
	OR_**1**_	95% CI	***P***-value	OR_**1**_	95% CI	***P***-value	OR_**1**_	95% CI	***P***-value
**Age**^**v1**^	1.01	0.99–1.04	0.32	1.02	1.01–1.04	0.01	1.03	1.01–1.05	0.002
**BMI**^**v1**^	0.95	0.94–0.97	< 0.001	0.96	0.95–0.97	< 0.001	0.98	0.96–0.99	< 0.001
**Gestational age at delivery**^**c**^	1.10	1.05–1.15	< 0.001	1.05	1.01–1.09	0.01	1.00	0.97–1.04	0.90
**Race or Ethnicity**^**v1**^									
Non-Hispanic white	**Reference**			**Reference**			**Reference**		
Non-Hispanic black	0.68	0.51–0.90	0.01	0.92	0.71–1.19	0.51	1.23	0.90–1.67	0.19
Hispanic	1.40	1.02–1.92	0.04	0.72	0.59–0.88	0.002	1.13	0.89–1.44	0.31
Other	1.30	0.87–1.95	0.20	0.86	0.69–1.09	0.21	0.99	0.77–1.28	0.94
**Poverty**^**v1**^									
> 200% of FPL	1.59	1.12–2.27	0.01	1.11	0.86–1.43	0.42	1.22	0.89–1.68	0.22
100–200% of FPL	1.47	1.03–2.10	0.03	1.09	0.83–1.43	0.55	1.52	1.08–2.13	0.02
< 100% of FPL	**Reference**			**Reference**			**Reference**		
Refused	0.85	0.62–1.14	0.27	0.65	0.49–0.86	0.003	0.88	0.62–1.26	0.49
**Education**^**v1**^									
Master’s degree and higher	2.81	1.90–4.14	< 0.001	1.89	1.51–2.36	< 0.001	1.08	0.84–1.40	0.53
Bachelor degree or less	2.12	1.62–2.76	< 0.001	1.43	1.20–1.70	< 0.001	1.05	0.85–1.30	0.66
High school or less	**Reference**			**Reference**			**Reference**		
**Planned pregnancy**^**v1**^									
Yes	1.09	0.86–1.37	0.48	1.48	1.27–1.73	< 0.001	1.18	0.98–1.42	0.08
No		**Reference**			**Reference**			**Reference**	
**Delivery by cesarean**									
Yes	0.93	0.75–1.17	0.55	0.88	0.76–1.02	0.10	1.10	0.93–1.30	0.28
No		**Reference**			**Reference**			**Reference**	
**Did you smoke any tobacco products in the month before delivery?**^**v4**^									
Yes		**Reference**			**Reference**			**Reference**	
No	2.32	1.56–3.45	< 0.001	2.04	1.19–3.45	0.01	3.12	1.43–7.14	0.01
**STAI-T**^**v1**^	0.985	0.975–0.996	0.01	0.990	0.983–0.998	0.01	0.997	0.988–1.006	0.47
**Random effect** Estimate (SE)	0.121 (0.07)			0.074 (0.04)			0.006 (0.01)		
**ICC**	0.04			0.02			0.002		

V1 (gestational age 6 weeks 0 days to 13 weeks 6 days), V2 (gestational age 16 weeks 0 days to 21 weeks 6 days), V3 (gestational age 22 weeks 0 days to 29 weeks 6 days), V4 (at time of delivery) – Variables obtained at Visits 1, 2, 3, 4 respectively

Model Area Under the Curve (AUC) reported as AUC (95% confidence interval)

*C* Variables obtained from chart abstraction, *FPL* Federal poverty level, *STAI-T* Spielberger State-Trait Anxiety Inventory, *ICC* Intra-class correlation

For women who breastfed, the odds of any breastfeeding for at least 6 months increased as maternal age increased (OR 1.03, 95% CI 1.01–1.04 for each year), while the odds of any breastfeeding for at least 6 months decreased with increasing BMI (OR 0.96, 95% CI 0.95–0.97, for each kg/m^2^, Table 3). Compared with non-Hispanic White women, Hispanic women who initially breastfed were less likely to breastfeed for at least 6 months (OR 0.73, 95% CI 0.59–0.89). Those with higher educational attainment, compared to those women completing high school or less, were more likely to breastfeed for at least 6 months (compared to high school or less- OR 1.89, 95% CI 1.51–2.36 for Master’s degree or higher; OR 1.43, 95% CI 1.20–1.70 for bachelor degree or less), as were those women who stated that the pregnancy was planned (OR 1.48, 95% CI 1.27–1.73). Women who did not smoke in the month prior to delivery were more likely to breastfeed for at least 6 months (OR 2.04, 95% CI 1.19–3.45). Women with higher anxiety scores were less likely to breastfeed for at least 6 months (OR 0.990, 95% CI 0.983–0.998 for each point increase on the scale).

Older age (OR 1.03, 95% CI 1.01–1.05), not smoking the month prior to delivery (OR 3.12, 95% CI 1.43–7.14), and being at 100–200% of the federal poverty level (OR 1.52, 95% CI 1.08–2.13) all were associated with higher odds of exclusive breastfeeding for at least 6 months. Increasing BMI was associated with lower odds of exclusive breastfeeding for at least 6 months (OR 0.98, 95% CI 0.96–0.99).

## Discussion

For women giving birth for the first time in a large and racially and ethnically diverse U.S. cohort, 89.8% breastfed their infant, with 58.2% breastfeeding for at least 6 months. Being older, having a lower BMI, not smoking in the month prior to delivery, and being at 100–200% of the federal poverty level (compared to those at < 100%) were associated with higher odds of exclusive breastfeeding for at least 6 months. While rates of breastfeeding and breastfeeding for at least 6 months differed across some racial and ethnic groups, the groups did not have significantly different odds of exclusive breastfeeding for at least 6 months.

Our findings are consistent with other reports noting that age and weight are associated with breastfeeding duration [26, 27]. We also found that women who smoked proximate to delivery were less likely to breastfeed. This finding was also noted among individuals in a Spanish birth cohort, in whom smoking was associated with a more than two-fold higher rate of formula feeding and shorter breastfeeding duration [28]. Previous studies found that other social markers (such as lower education attainment and not attending prenatal classes), as well as physiologic factors such as delayed onset of lactation, inadequate milk production, nipple pain, latching problems and lack of social support are associated with early discontinuation of breastfeeding [29, 30]. Attending prenatal classes and a previous successful breastfeeding experience have been associated with longer duration of breastfeeding [11, 30].

We found that Hispanic women were more likely to initiate breastfeeding, but that these women were less likely to breastfeed for at least 6 months. This has also been noted in a few other studies [31–33]. Level of acculturation and whether women speak primarily English or Spanish may impact breastfeeding duration in Hispanic women [33, 34]. Spanish-speaking women may represent a group who could benefit from culturally-sensitive, family-level interventions and work place policies to increase breastfeeding duration [33, 35]. While non-Hispanic Black women giving birth for the first time in the cohort had lower odds of initiating breastfeeding, those who did breastfeed had similar odds of exclusively breastfeeding for at least 6 months. Thus, improved initial uptake of breastfeeding for non-Hispanic Black mothers is important.

Multiple reports have addressed some aspects of the relationship of maternal psychosocial measures and mental health on breastfeeding duration. As in our cohort, other smaller cohorts noted that higher anxiety scores are independently associated with shorter breastfeeding duration [36–39]. Social support was also positively associated with breastfeeding duration [27]. Other studies that found a negative association of depression and breastfeeding highlighted the importance of a pre-pregnancy diagnosis of depression [40]. In the current cohort, a history of mental health disorders was not significantly associated with breastfeeding duration. Only a history of bipolar disorder was associated with lower rates of breastfeeding, but with so few women endorsing that history (*n* = 79), these findings should be interpreted with caution as it was not included in the regression model.

Breastfeeding benefits are numerous for both the mother and infant. Benefits to women who breastfeed include increased caloric expenditure in the postpartum period and lower risks for cardiovascular disease, type 2 diabetes, and breast cancer [2, 6, 9]. Breastfed infants have decreased long-term risks of childhood cancers and many other positive health benefits [41–43]. Formula costs approximately $1200–$1500 USD per year per infant which places extra financial burden on parents and community resources [44]. These individual medical and nonmedical benefits translate into significant population-level benefits given the nearly 4 million births that occur yearly in the US [10, 45].

Investigators and health agencies have called for identifying modifiable factors that can be targeted to help improve breastfeeding duration. The need for improvements is underscored by the findings in the current study: the rate of exclusive breastfeeding for at least 6 months (24.7%) was well below the 2025 WHO goal of 50% [11]. The findings in our population are slightly lower than other published rates in the US [12]. This may be because our population only included women giving birth for the first time. Educational campaigns and community-level support have been proposed to help improve breastfeeding rates, particularly among underserved populations [11, 46]. Identifying the women most at risk for not initiating any breastfeeding may be an important approach to help target intervention strategies as well. A comprehensive approach of support includes not only education and engagement, but also policies that foster and protect a woman’s right, choice, and opportunity to breastfeed [11, 47, 48].

Our study was limited in that we did not ask women for a specific date or number of weeks that they breastfed. The first interval contact was no earlier than 6 months after delivery, allowing for women who had breastfed for at least 6 months to note that outcome. As we asked the women to answer the question after breastfeeding may have stopped, it is possible that their responses may have been affected by recall or social-desirability bias. Yet, it has been shown that most women have accurate recall of duration of breastfeeding their first baby [49, 50], and accordingly we do not believe this type of bias is likely to influence our results systematically. We also did not ask participants when they returned to work, which may influence breastfeeding longevity [51, 52]. We also did not address other reasons for discontinuation, such as perception of milk supply, in the current study. We were unable to collect information on contraception or other medications being used in the postpartum timeframe. As a multicenter, diverse cohort, our findings are generalizable to similar populations of individuals giving birth for the first time.

Designing multilevel public health strategies and policies to help optimize prepregnancy health and education regarding breastfeeding, along with support in the early postpartum period, may help improve the rate of exclusive breastfeeding for at least 6 months in order to reach the 2025 Global Nutrition Target of at least 50% of mothers. These programs may include smoking cessation programs, more frequent contact with lactation specialists in the early postpartum period, community-based peer support for women in at-risk racial groups or with high anxiety, and effective workplace policies to support breastfeeding for women returning to work [53–56]. Supporting women who begin breastfeeding but who may stop before 6 months could be a particular area of focus, particularly for Hispanic women as seen in this cohort. These strategies and interventions, which help to ensure women who want to breastfeed are able to continue, should be tested in prospective cohorts as the benefits of breastfeeding extend to the infant, the mother, and society.

## Conclusion

In conclusion, in this cohort of women giving birth for the first time, exclusive breastfeeding for at least 6 months was associated with older age, lower BMI, and not smoking in the month before delivery.

### Supplementary Information

**Additional file 1.** Interval contact form. This is one of the pages of the Interval Contact Form used for contacting participants in the nuMoM2b-HHS that asked about feeding of the infant after birth.

## Data Availability

Data from nuMoM2b used for this analysis are available in the NICHD DASH repository (https://dash.nichd.nih.gov/). The data on breastfeeding longevity from the nuMoM2b-HHS are not yet publicly available. Requests for these data will be considered by the Data Coordinating Center, Research Triangle International. Contact corresponding author for details.

## Appendix

Table 4

**Table 4:** Correlation coefficients of collinearity for psychosocial measures in the cohort

Pearson Correlation Coefficients, N = 5249 Prob > |r| under H0: Rho = 0						
	EDIN_DEPV3	MS_PSS	CD_RISC	STAI_T	COHEN_PSSV3	hassleINT_ratio_PES
**EDIN_DEPV3** Edinburgh depression - V3	1.00000	−0.17221	−0.41031	0.58379	0.73124	0.40899
		<.0001	<.0001	<.0001	<.0001	<.0001
**MS_PSS** Perceived Social Support - V1	− 0.17221	1.00000	0.20530	− 0.26338	− 0.17336	− 0.17259
	<.0001		<.0001	<.0001	<.0001	<.0001
**CD_RISC** Connor–Davidson Resilience - V2	− 0.41031	0.20530	1.00000	−0.54719	− 0.43664	− 0.32266
	<.0001	<.0001		<.0001	<.0001	<.0001
**STAI_T** Spielberger Trait Anxiety - V1	0.58379	−0.26338	− 0.54719	1.00000	0.58878	0.40869
	<.0001	<.0001	<.0001		<.0001	<.0001
**COHEN_PSSV3** Cohen Perceived Stress - V3	0.73124	−0.17336	−0.43664	0.58878	1.00000	0.40674
	<.0001	<.0001	<.0001	<.0001		<.0001
**hassleINT_ratio_PES** Pregnancy hassles/uplifts, intensity ratio - V3	0.40899	−0.17259	−0.32266	0.40869	0.40674	1.00000
	<.0001	<.0001	<.0001	<.0001	<.0001	

V1 (gestational age 6 weeks 0 days to 13 weeks 6 days), V2 (gestational age 16 weeks 0 days to 21 weeks 6 days), V3 (gestational age 22 weeks 0 days to 29 weeks 6 days), V4 (at time of delivery) – Variables obtained at Visits 1, 2, 3, 4 respectively

*EPDS* Edinburgh Postnatal Depression Scale, *MSS* Multidimensional Perceived Social Support Scale, *CD-RISC* Connor-Davidson Resilience Scale, *STAI-T* Spielberger State-Trait Anxiety Inventory, *PSS* Perceived Stress Scale, *PES* Pregnancy Experience Scale

## Abbreviations

aOR: Adjusted Odds Ratio
AUC: Area under the curve
BMI: Body mass index
CI: Confidence interval
CD-RISC: Connor-Davidson Resilience Scale
EPDS: Edinburgh Perinatal Depression Scale
FPL: Federal poverty level
HHS: Heart Health Study
MSS: Multidimension perceived social support scale
NICHD DASH: National Institute of Child Health and Human Development Data and Specimen Hub
nuMoM2b: Nulliparous Pregnancy Outcomes Study: monitoring mothers-to-be
OR: Odds ratio
PES: Pregnancy Experiences Scale
PSS: Perceived Stress Scale
SD: Standard deviation
STAI-T: Spielberger State-Trait Anxiety Inventory
USD: United States dollars
WHO: World Health Organization
