# Supplementary material for: Factors associated with duration of breastfeeding in women giving birth for the first time
Source: BMC Pregnancy Childbirth. 2022 Sep 22;22:722. doi: 10.1186/s12884-022-05038-7 (PMC9494803; doi:10.1186/s12884-022-05038-7)
Supplement: Supplementary file 1 — Additional file 1. Interval contact form. This is one of the pages of the Interval Contact Form used for contacting participants in the nuMoM2b-HHS that asked about feeding of the infant after birth. [file 12884_2022_5038_MOESM1_ESM.pdf]

Study ID	Staff ID	Date Interview Completed
__ __ __ __ __ __ __ __ __	__-__-__ __ __	__ __ / __ __ / __ __ __ __ (mm dd yyyy)

D. nuMoM2b Offspring Questions ***[FOR T08, READ THE INTRODUCTION, SKIP #D1 THROUGH #D3, AND ASK REMAINING ITEMS IN SECTION D ONLY IF THE nuMoM2b BABY IS KNOWN TO BE ALIVE. OTHERWISE SKIP TO SECTION E.]***

First, we want to ask some questions about the baby you delivered at the end of your nuMoM2b pregnancy. We are not asking about any babies that you may have had since then.

1. Did you ever breastfeed this baby?
   - Yes ☐01 → **CONTINUE TO #D1a**
   - No ☐02 → **SKIP TO #D2**
   - Don't know ☐-8 → **SKIP TO #D2**

   IF YES:

   a) Was there a period of time when you fed this baby only breast milk (no formula, milk, juice, or food)? This is called exclusive breastfeeding.
   - Yes ☐01 → **CONTINUE TO #D1a1**
   - No ☐02 → **SKIP TO #D1b**
   - Don't know ☐-8 → **SKIP TO #D1b**

   IF YES:

   1) About how old was this baby when exclusive breastfeeding stopped?
   - Less than 6 weeks ☐01
   - 6 weeks to 11 weeks ☐02
   - 3-6 months ☐03
   - More than 6 months ☐04
   - Still exclusively breastfeeding ☐05
   - Don't know ☐-8

   b) About how old was this baby when all breastfeeding stopped?
   - Less than 6 weeks ☐01
   - 6 weeks to 11 weeks ☐02
   - 3-6 months ☐03
   - More than 6 months ☐04
   - Still breastfeeding ☐05
   - Don't know ☐-8

2. Has this baby started eating solid foods, for example cereal?
   - Yes ☐01 → **CONTINUE TO #D2a**
   - No ☐02 → **SKIP TO #D3**
   - Don't know ☐-8 → **SKIP TO #D3**

   IF YES:

   a) About how many months old was this baby when you first began feeding solid foods? |__|__| months

3. How much did your baby weigh the last time weight was measured?

   a) |__|__|.|__| kilograms ***OR*** b) |__|__|__| pounds

   c) About how many months old was this baby when this weight was taken? |__|__| months

4. During the last 12 months, on average, what amount of their time did this child usually spend with you?

   a) |__|__|__| % of time ***OR*** b) |__|__|.|__| hours per day ***OR***

   c) Don't know ☐-8 ***OR*** d) Refused ☐-7

IF D4a=0 OR D4b=0 THEN SKIP TO Section E. ELSE CONTINUE.

5. Over the last 12 months, thinking about the time you spend with this child, do you think you spent too much time with your child, too little time, or about the right amount of time?
   - Too much time ☐01
   - Too little time ☐02
   - About the right amount of time ☐03
   - Don't know ☐-8
   - Refused ☐-7

6. In general, over the last 12 months, how would you describe this child's health?
   - Excellent ☐01
   - Very good ☐02
   - Good ☐03
   - Fair ☐04
   - Poor ☐05
   - Don't know ☐-8
   - Refused ☐-7
